# Lethal Dose, Clinical Signs, Gross and Microscopic Lesions Induced by *Aeromonas veronii* Biovar *sobria* A4 Strain in Experimentally Challenged Nile Tilapia (*Oreochromis niloticus*)

**DOI:** 10.1155/vmi/5525701

**Published:** 2025-06-12

**Authors:** Joseph M. Ndegwa, Isaac R. Mulei, Lucy W. Njagi, Philip N. Nyaga, Daniel W. Wanja, Shimaa E. Ali, Jérôme Delamare-Deboutteville

**Affiliations:** ^1^Department of Veterinary Pathology, Microbiology and Parasitology, Faculty of Veterinary Medicine, University of Nairobi, P.O. Box 29053-00625, Kangemi, Nairobi, Kenya; ^2^Department of Veterinary Pathology, Microbiology and Parasitology, Faculty of Veterinary Medicine and Surgery, Egerton University, P.O. Box 536-20115, Njoro-Egerton, Njoro, Kenya; ^3^Department of Hydrology, Veterinary Research Institute, National Research Centre, Dokki, Giza 12622, Egypt; ^4^World Fish Headquarters, Jalan Batu Maung, Batu Maung, Bayan Lepas 11960, Penang, Malaysia

**Keywords:** *Aeromonas veronii* biovar *sobria* A4 strain, clinical sign, gross lesions, histopathological lesions, lethal dose (LD_50_)

## Abstract

*Aeromonas veronii* biotype *sobria* is a potential aquatic zoonotic pathogen and a major cause of freshwater bacterial infections in cultured fish globally, leading to substantial economic losses. This study aimed to establish the median lethal dose (LD_50-96 h_) for *Aeromonas veronii* biovar *sobria* A4 strain and to demonstrate induction of clinical signs, gross and microscopic lesions in experimentally infected juvenile Nile tilapia (*Oreochromis niloticus*). *Aeromonas veronii* biotype *sobria* A4 strain used in this study were obtained from water samples from ponds with high fish mortality at Cavarino farm in Narok County, Kenya. Six groups each comprising 10 fish were intraperitoneally injected with 0.1 mL of *A. veronii* biovar *sobria* A4 strain suspension at: 1.5 × 10^4^, 1.5 × 10^5^, 1.5 × 10^6^, 1.5 × 10^7^, 1.5 × 10^8^, and 1.5 × 10^9^ colony forming units per mL (CFU/mL) respectively and the bacteria was afterward recovered from kidney and hepatopancreas of freshly dead fish. Duplicate control groups (each *n* = 10) were injected with sterile physiological saline before the lethal dose group were injected with varying concentration of the *A. veronii* biovar *sobria* A4 strain. The LD_50-96 h_ of *A. veronii* biovar *sobria* A4 strain was found to be 1.5 × 10^8^ CFU/mL. Clinical signs and gross lesions observed in the lethal dose group were: skin hemorrhages (20%), erosion of the fins including caudal fin with scale loss exposing underlying skin (13.7%), congested and hemorrhagic gills (15%), hepatic hemorrhages and enlargement (21.3%), distension of gall bladder (18.8%), splenomegaly and congestion (22.5%), and ascites (16.3%). The main histopathological lesions observed in the gills were focal hemorrhages, atrophy of the filaments and loss of lamellae in some filaments with mononuclear cellular infiltration; on the liver there were; hemorrhages, infiltration with lymphocytes and melanomacrophages, degenerative hepatocytes and focal necrosis. There was extensive hemosiderosis with increased melanomacrophages in the spleen. The kidney showed extensive hemorrhages, localized coagulative necrosis, atrophied glomeruli and multifocal mononuclear cellular infiltration in the interstitium. The findings will lay a foundational basis for subsequent investigations into the host-pathogen interaction, therapeutic approaches, and epidemiology of *Aeromonas veronii* biovar *sobria*.

## 1. Introduction

Freshwater aquaculture significantly contributes to world's economies and food production, livelihood and nutritional supply especially in developing countries including Kenya [1]. An extensive array of fresh-water creatures has been reared with tilapia, catfish and carp being the most species reared worldwide [1, 2]. However, the aquaculture sector in Kenya is faced by various challenges such as diseases caused by bacteria [3], fungi [4], viruses [5], and parasites [6, 7].

Bacterial infections among fish are the most crucial in causing massive economical deprivations worldwide in fish industry [8]. This is also the situation with aquaculture production in Kenya [3]. A considerable upsurge in the number of bacterial species believed to cause diseases in fish has been witnessed. About 125 species of bacteria of 34 families have been established as contributing agents of fish diseases worldwide [9]. New findings have revealed that several infective bacteria, such as *Flavobacterium columnare, Pseudomonas* spp*., Aeromonas hydrophila, Streptococcus iniae, Edwardsiella tarda*, and *Citrobacter freundii*, among others, are the aetiological agents of fish bacterial diseases [10].

*Aeromonas* species are majorly found in a wide array of surroundings including saltwater, estuarine and freshwater environments [11–13]. They are also capable of causing a disease to an extensive variety of hosts apart from fish such as mammals, reptiles and amphibians [13, 14]. *Aeromonas* species also ubiquitously occur in water surroundings and they cause fish diseases [15]. They are also linked with human wound and gastrointestinal contagions [13].

*Aeromonas veronii* has been recognized as the etiologic mediator mainly causing fish death globally amongst the 31 types in genus *Aeromonas* [16]. It is similarly linked by epizootic ulcerative pattern in reared fish types [16, 17]. *Aeromonas veronii* is a devious organism which is able to produce an ailment in a debilitated fish stocks or as minor attackers in fish population suffering from other sicknesses [18]. It has an extensive host predisposition extended from *Schizothorax prenanti, Cyprinus carpio*, *Ictalurus punctatus, Anguilla japonica*, medicinal leech, *Oreochromis niloticus, Andrias davidianus*, to humans being. There are two distinguishable biovars of *Aeromonas veronii* namely; biovar *sobria* and biovar *veronii* [19]. Biotype *sobria* is the rifer bacterium of the two in fish; causing dermal ulceration.

This organism has robust capability to acclimatize to the outside setting and produce numerous virulence features which lead to different ailments [20, 21]. Altogether, *Aeromonas veronii* has been deliberated as the organism which needs instant consideration as it has been affecting numerous reared fish species. The objective of this study was to establish the lethal dose (LD_50-96 h_) of *Aeromonas veronii* biovar *sobria* A4 strain, the clinical signs, gross lesions and microscopic lesions induced in *Oreochromis niloticus* after intraperitoneal injection of the organism.

## 2. Materials and Methods

### 2.1. Ethical Approval

All procedures for experimentations concerning live fish done in this study were permitted by the Biosafety, animal use and ethics committee for experimentations on live non-human vertebrates, Faculty of Veterinary Medicine, University of Nairobi, Kenya. (REF: FVM BAUEC/2023/424).

### 2.2. Source of *Aeromonas veronii* Biovar *sobria* Isolate

Isolates of *Aeromonas veronii* biovar *sobria* A4 strain utilized in this study were recovered from pond water samples from Cavarino farm located in Narok county, Kenya. The farm practices mixed farming of pig production, layer-chicken rearing, fish farming (Nile tilapia, ornamental fish and catfish), beef feedlot and crop farming. There were mass mortalities exceeding 10% in all the fish species and age groups of fish reared in this farm reported in May 2022 with unknown aetiology. The fish presented with hemorrhages and ulceration on the skin. Water samples from the affected fish ponds was collected in sterile universal bottles and submitted to the bacteriology laboratory, department of Veterinary Pathology, Microbiology and Parasitology, University of Nairobi, for bacteriological analysis, as described by Ndegwa et al. [22].

Briefly, a loopful of pond water sample was directly streaked aseptically onto freshly prepared Nutrient agar and Blood agar and incubated for 24 h at 37° C. The colonies were then subjected to routine bacteriological identification methods such as Gram staining, indole test, methyl red test, Voges–Proskauer (VP) test, catalase and oxidase test, and sugar utilization of glucose, lactose, sucrose and mannitol. Pure colonies were stored in nutrient agar slant and tryptone soy broth. Presumptive *A. veronii* (identified using biochemical methods) was then confirmed to be *A. veronii* biovar *sobria* using BD Phoenix automated identification (USA) and whole genome sequencing. Whole genome sequences of *Aeromonas veronii* biovar *sobria* A4 strain was then deposited in the GenBank database under the accession number, SAMN43496132 in the NCBI databases available at; https://www.ncbi.nlm.nih.gov/biosample/SAMN43496132. The confirmed *Aeromonas veronii* biovar *sobria* A4 strain isolates were preserved in tryptose soya broth supplemented with 30% glycerol at −80°C.

### 2.3. Fish and Acclimatization

The trial fish were bought from a viable fish farm and acclimated in aquaria for 2 weeks preceding experimental challenge. The fish were fed on commercially formulated feed for these species (fat 13%, fiber 1.7%, ash 8.0%, phosphorous 1.2%, calcium 2.0%, methionine + cysteine 2.3%, lysine 3.5%, vitamins) at 3% of their body weight. The fish were fed by ad satiation at 3-4 times a day.

### 2.4. Preparation of the *Aeromonas veronii* Biovar *sobria* A4 Strain Suspension Used as Inoculum

Previously preserved stock cultures of *A. veronii* biovar *sobria* A4 strain were revived in Tryptic Soy Agar (TSA) and then incubated at 37° C for 24 h. The colonies were emulsified in freshly prepared sterile physiological saline, until visible turbidity matched the turbidity of a McFarland Standard 0.5. The bacterial suspension was then diluted in tenfold using sterile physiological saline to obtain a cell density of 1.5 × 10^4^, 1.5 × 10^5^, 1.5 × 10^6^, 1.5 × 10^7^ and 1.5 × 10^8^ [23].

### 2.5. Experimental Design

The experimental study was carried out in the wet laboratory at the department of Veterinary Pathology, Microbiology and Parasitology, Faculty of Veterinary Medicine, University of Nairobi; where a total of 80 healthy postfingerling Nile tilapia (*Oreochromis niloticus*) with an average body weight of 15 g was used. The fish were distributed into 8 assemblages with 10 fish per set and distributed into 8 glass aquaria (60 × 50 × 40 cm), occupied by water up to a height of 30 cm. The first six groups of fish were intraperitoneally injected with varying concentration of *Aeromonas veronii* biovar *sobria* A4 strain suspension; 1.5 × 10^4^, 1.5 × 10^5^, 1.5 × 10^6^, 1.5 × 10^7^, 1.5 × 10^8^, and 1.5 × 10^9^ CFU/mL. The control group in duplicate were inoculated with sterile physiological saline.

### 2.6. Determination of Median Lethal Dose (LD_50-96_) of *Aeromonas veronii* Biovar *sobria* A4 Strain

Fish were first anaesthetized using MS222 at a concentration of 150 μg/L [24] before intraperitoneal injection of 0.1 mL of the bacterial suspension at 1.5 × 10^4^, 1.5 × 10^5^, 1.5 × 10^6^, 1.5 × 10^7^, 1.5 × 10^8^ and 1.5 × 10^9^ CFU/mL using a gauge 26 needle fitted on 1 mL syringe. The control assemblage received an intraperitoneal injection of an equivalent measurement of physiological saline (0.85% sodium chloride). The fish were monitored for clinical signs and mortalities for 10 days postinfection three times per day. The LD_50_ was estimated using Reed and Muench method [25].

(1) $$\begin{array}{c} \text{Proportionate distance}=\frac{\left[\left(\text{mortality at dilution next above }50\%\right)-50\%\right]}{\left[\left(\text{mortality next above }50\%\right)-\left(\text{mortality next below }50\%\right)\right]},\\ \log\;{\text{LD}}_{50}=\text{Dilution above }50\%-\left(\text{Proportionate distance}\times \text{Dilution factor}\right). \end{array}$$

Thus;

(2) $$\begin{array}{c} \text{Proportionate distance}=\left[\frac{\left(0.55-0.5\right)}{\left[0.55-0.3043\right]}\right.\\ =\frac{0.05}{0.2457}\\ =0.2035,\\ \log\;{\text{LD}}_{50}={1.5\times 10}^{8}-\left(0.2035\times 0.1\right)\\ ={1.5\times 10}^{8}-0.02035\\ =1.4999999997965\times 10^{8}\\ \approx 1.5\times 10^{8}. \end{array}$$

### 2.7. Postmortem Examination

Necropsy was done according to Noga [26]. Gross variations in each of core organ were defined and recorded. The dissecting kit was first autoclaved and allowed to cool before dissecting the fish samples. An incision was made along the ventral midline through the pelvic girdle and almost to the vent. The anterior peritoneal lining under the pectoral girdle was then cut and an incision made starting above the pectoral fin to remove the lateral body wall and reveal the internal viscera. The operculum was also excised to expose the gills. All the identified gross lesions were documented.

### 2.8. Histopathological Evaluation

Gills, kidney, spleen and liver were obtained from moribund and freshly dead fish. They were fixed in 10% neutral-buffered formalin for at least 24 h before processing using paraffin-embedded technique. The tissues were dehydrated in graded alcohol at 50%, 70%, 80%, 90% and absolute alcohol followed by clearing using xylene. They were sectioned at 4 μm and stained with haematoxylin-eosin (H&E). The slides were examined under × 10, × 40, and × 100 (oil immersion) objective lenses. Lesions were identified, and photomicrographs were captured using the 14-megapixel Amscope Aptina Digital Camera MU1400 series. The histopathological findings were recorded in a Microsoft Excel database for analysis.

### 2.9. Re-Isolation of *Aeromonas veronii* Biovar *sobria* A4 Strain

Standard necropsy procedures of freshly dead and/or moribund fish were carried out as described by Noga [26]; followed by aseptic sampling of kidney and hepatopancreas samples for bacteriological analysis for re-isolation of *A. veronii* biovar *sobria* A4 strain. The samples were streaked onto TSA, blood agar and MacConkey agar plates to confirm the actual cause of death and rule out possibility of other factors that might be playing a part in the death of the experimental fish.

### 2.10. Water Quality Assessment During *A. veronii* Biovar *sobria* A4 Strain Inoculation Assays

Water quality parameters were maintained according to American Public Health Association (APHA) standards. On a daily basis, unconsumed feed and fish wastes (fecal matter) was removed by siphoning; thereafter a percentage of the capacity of water in the aquaria was changed. Dissolved oxygen (DO), pH and water temperature were measured and noted daily using HANNA HI98130 instrument.

### 2.11. Statistical Analysis

Water quality values were expressed as mean ± SD. Cumulative mortality data (%) were presented as mean ± standard error and graphically using GraphPad Prism. Fish survival rates against *A. veronii* biovar *sobria* A4 strain challenge were compared via One-way ANOVA with significance at *p* < 0.05. All analyses used GraphPad Prism.

## 3. Results

### 3.1. Mortalities of *Oreochromis niloticus* Infected With *Aeromonas veronii* Biovar *sobria* A4 Strain

**Figure 1:**
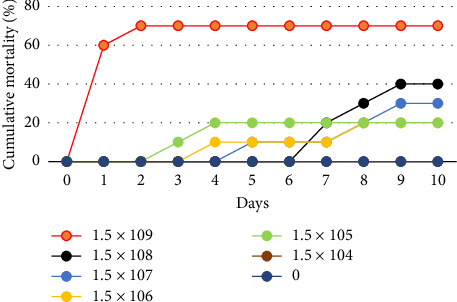
Cumulative mortality curves of Nile tilapia fish after intraperitoneal inoculation with *A. veronii* biovar *sobria* A4 strain.

**Figure 2:**
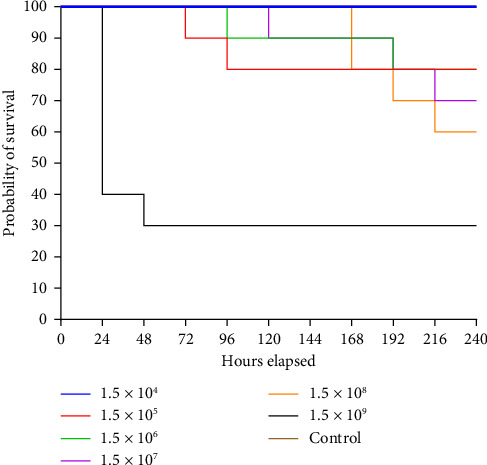
Survival rate curves of Nile tilapia after intraperitoneal inoculation with *A. veronii* biovar *sobria* A4 strain.

The survival rate of *O. niloticus* experimentally infected with *A. veronii* biovar *sobria* illustrated in Figures 1, 2 and Table S1. The outcomes showed that 60% mortality of *O. niloticus* occurred within the first 24 h in *O. niloticus* inoculated with 1.5 × 10^9^ CFU/mL. The mortalities within 240 h period were 20%, 20%, 30%, 40% and 70% of fish inoculated with dilutions 10^5^ to 10^9^, respectively. However, no death was recorded after 240 h/10 days of injection with 1.5 × 10^4^ CFU/mL. Similarly, there were no deaths nor clinical signs observed in the control groups. It was determined that the lethal dose of *Aeromonas veronii* biovar *sobria* in Nile tilapia was 1.5 × 10^8^ CFU/mL.

Moreover, the results of water quality assessment during *A. veronii* biovar *sobria* A4 strain inoculation assays showed that the DO, water temperature and pH ranged between 5.6 ± 0.2, 26.11 ± 1.29°C and 7.62 ± 0.25 respectively.

### 3.2. Clinical Signs and Gross Lesions Observed in Nile Tilapia Challenged With *A. veronii* Biovar *sobria* A4 Strain

**Figure 3:**
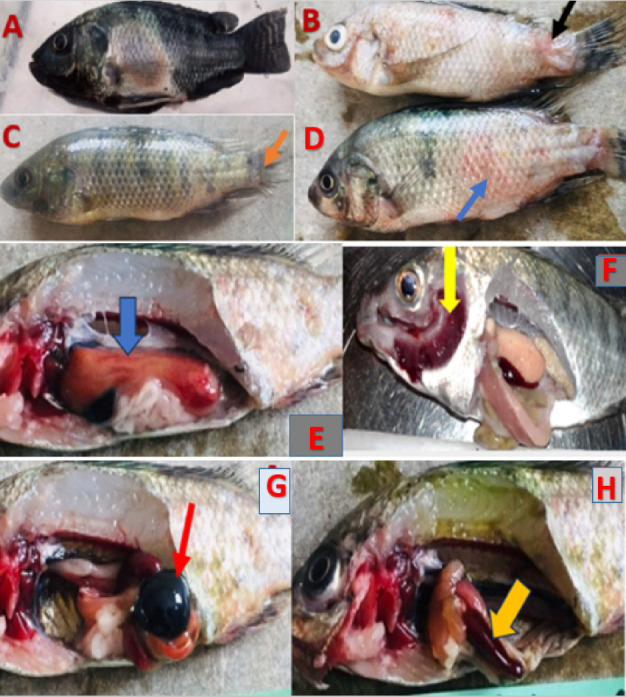
Clinical signs and pathological changes of nile tilapia intraperitoneally inoculated with A. *veronii* biovar *sobria* A4 strain. (A) Darkening of the skin surface of the fish. (B) Ulcerations at the base of caudal fin (arrow). (C) Erosion of caudal fin (arrow). (D) Hemorrhages on the skin surface (arrow). (E) Enlarged and hemorrhagic liver (arrow) (F) congested and hemorrhagic gills (arrow). (G) Distended gall bladder (arrow). (H) Enlarged and congested spleen (arrow).

The main gross lesions that were observed in most of moribund and dead fish were; darkening of the skin (Figure 3(a)), scale loss exposing the underlying skin at the base of caudal fin (Figure 3(b)), erosion of the fins (Figure 3(c)), hemorrhages over the body surface (Figure 3(d)), hepatomegaly and liver hemorrhages (Figure 3(e)), ascites, opacity of the eye, exophthalmia, amplified mucus on the skin surface, congestion on the operculum, congested and hemorrhagic gills (Figure 3(f)), distended gall bladder (Figure 3(g)), enlargement and congestion of spleen (Figure 3(h)), and inflamed vent.

The fish challenged with *A. veronii* biovar *sobria* A4 strain showed symptoms like erratic swimming behaviour with loss of balance and swimming at the bottom of the aquarium, reduced feed consumption, irregular breathing and lethargy. These signs were not observed in control groups.

**Figure 4:**
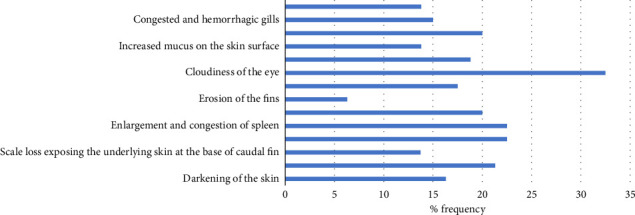
Frequency of pathological lesions in assay of inoculation of nile tilapia fish with *A. veronii* biovar *sobria* A4 strain.

**Figure 5:**
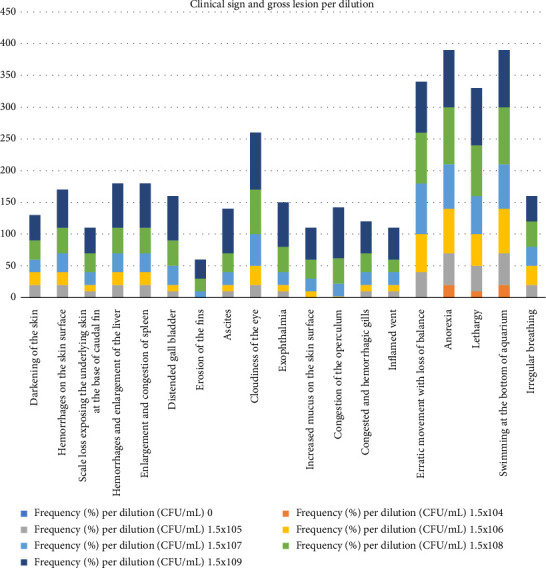
Macroscopic lesions and clinical signs per dilution following inoculation of nile tilapia with *A. veronii* biovar *sobria* A4 strain.

Frequency (%) of pathological lesions in assay of inoculation of tilapia fish with *A veronii* biovar *Sobria* is shown in Figure 4 and Table S2. Frequency of clinical signs and gross lesions per dilution is shown in Figure 5 and Table S3.

### 3.3. Re-Isolation of *Aeromonas veronii* Biovar *sobria* A4 Strain

Pure colonies with colonial morphology similar to *Aeromonas veronii* biovar *sobria* were observed to grow in all the media. The re-isolated bacterium was Gram negative, catalase and oxidase positive, indole positive, methyl red negative, VP positive, citrate positive, urea negative, and utilized glucose, sucrose and mannitol but not lactose.

### 3.4. Histopathological Changes in Nile Tilapia (*Oreochromis niloticus*) Experimentally Challenged With *A. veronii* Biovar *sobria* A4 Strain

**Figure 6:**
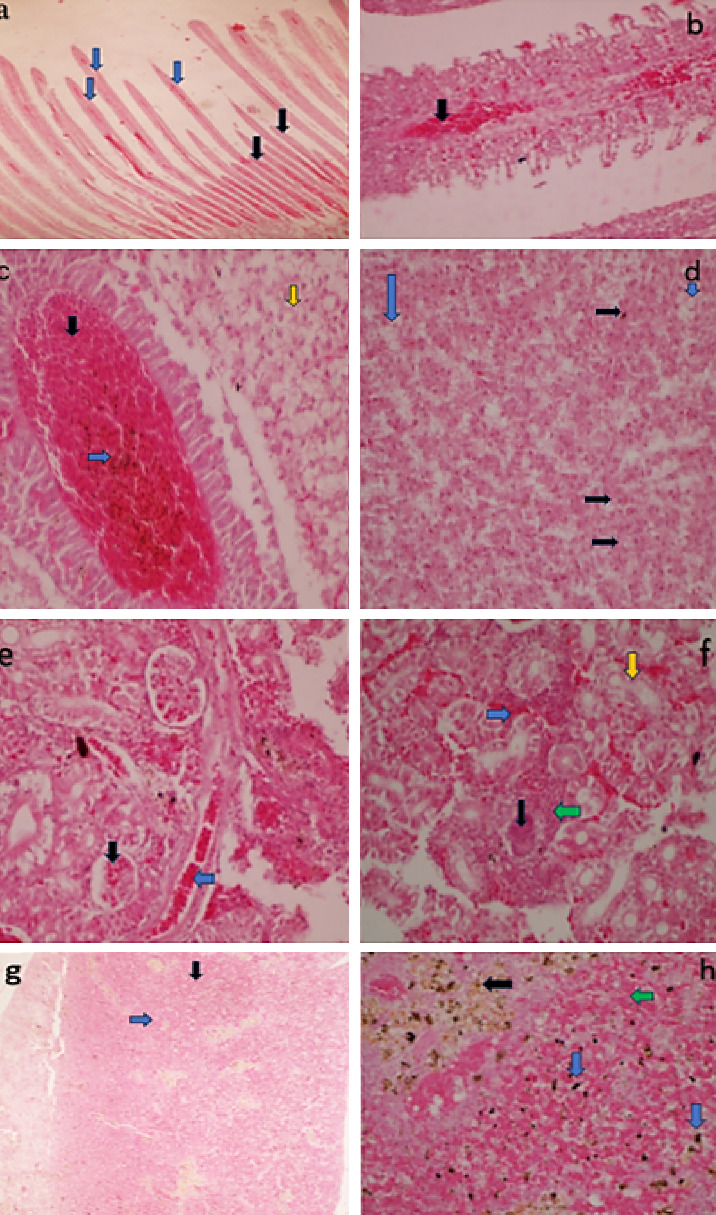
Histopathological changes of Nile tilapia inoculated with *A. veronii* biovar *sobria*; (a) loss of lamellae (blue arrow) and atrophy of filaments (black arrow) in the gills (× 40) [H&E]. (b) Congestion in the gills (black arrow) (× 400) [H&E]. (c) Congestion (black arrow), melanomacrophages (blue arrow) and coagulative necrosis (orange arrow) in the liver (× 400) [H&E]. (d) Acute hepatocyte swelling (black arrow) and vacuolar degeneration (blue arrow) in the liver (× 400) [H&E]. (e) Atrophied glomerular (black arrow) and congestion (blue arrow) in the kidney (× 400) [H&E]. (f) Cast (black arrow), interstitial hemorrhages (blue arrow), multifocal mononuclear cellular infiltration in the interstitium (green arrow) and degenerating tubules (orange arrow) in the kidney (× 400) [H&E]. (g) Increased red pulp (black arrow) and depleted lymphoid follicles (blue arrow) in the spleen (× 40) [H&E]. (h) Melanomacrophages (blue arrow), hemosiderosis (black arrow) and increasing red pulp with decreasing white pulp (green arrow) in the spleen (× 400) [H&E].

Experimentally infected fish exhibited frequent and intense pathological lesions in all organs evaluated. The PBS treated groups showed no histopathological changes. The control group was used to compare histopathological changes in the organs of bacterial challenged groups. The main changes observed in gills, kidney, liver and spleen are given in Figure 6.

## 4. Discussion

The main clinical signs in experimental fish infected with *Aeromonas veronii* biovar *sobria* A4 strain were similar to those reported in previous studies by Eissa et al. [27], Jagoda et al. [28], Cai et al. [29] and Wahli et al. [30] confirming the first reporting of such a finding in Kenya. The erratic movements with loss of balance and irregular breathing observed could be as a result of acetylcholinesterase produced by this bacterium which displays narcotic effects by affecting the central nervous system of fish [31].

There were cumulative deaths with increased bacterial concentration, since more toxins would be released by the bacteria that could harm the core organs of the fish [32]. Diseased fish died as early as 24 h (day 1) and as late as 216 h (day 9), indicating the ability of *Aeromonas veronii* biovar *sobria* to cause acute-to-chronic infection in fish. This was similar to studies carried out by Chen et al. [33] and Aly et al. [34]. In addition, *A. veronii* biovar *sobria* A4 strain was recovered from the diseased fish establishing Koch's Postulates. This signifies that this bacterium is pathogenic.

In this study, deaths were observed as early as 24 h postinfection in the highest bacterial concentration. In the same way, acute mortality was similarly found in the investigational test of red tilapia (*Oreochromis* species) with *A. veronii* in a previous study [35]. However, there was no 100% mortality even in the highest concentration similar to a study done of *A. hydrophila* in the giant fish from Amazon (*Arapaima gigas*), by Dias et al. [11] where there was 86% mortality in 10^10^ CFU/mL concentration. Also, the early mortalities observed indicates that the contagion produced by this bacterium has a rapid development period [35]. Comparable phenomena in tongue soles disease-ridden with a pathogenic *Shewanella algae* were described by Han et al. [36], who distinguished that the brief development period caused a rapid disease development.

Extracellular enzymes and toxins accompanied with some structural features of this pathogen, are thought to be significant aspects in its virulence and these include cytotonic and cytotoxic enterotoxins, leucocidins, proteases, lipases, and hemolysins [37]. The bacteria that produce toxins (aerolysin, cholinesterase, enterotoxins, hemolysins, proteases, adhesins and haema-glutinins, etc.) are fatal to fish when in high amount [31, 38, 39]. These virulence factors might be the ones responsible for the clinical signs and gross lesions observed which included hemorrhages on the body surface of fish, ulceration at the base of caudal fins, congestion and hemorrhages of internal organs and ascites, among others.

The LD_50_ for *Aeromonas veronii* biovar *sobria* A4 strain in this study was estimated to be 1.5 × 10^8^ CFU/mL. This was nearly comparable to research conducted by Aly et al. [34] in which the LD_50_ for *Aeromonas veronii* biovar *sobria* was 1.5 × 10^7^ CFU/mL in Nile tilapia (*Oreochromis niloticus*). However, this was different from a study of *A. veronii* in *Carassius auratus gibelio* (Crucian carp) conducted by Chen et al. [33] in which the LD_50_ was 1.31 × 10^7^ CFU/mL. This could be due to species difference in which different species respond differently to infection by a bacterium pathogen [40]. Conferring to the principles described by Mittal et al. [41], a bacteriological strain can be described as ascetically infectious if LD_50_ values are within the range of 10^6^–10^7^ CFU/g of fish body weight. In this study, however, the LD_50_ of *A. veronii* biovar *sobria* was calculated to be 1.5 × 10^8^ CFU/mL which therefore shows that it is vastly virulent to Nile tilapia (*Oreochromis niloticus*).

The histopathological changes observed were comparable to those reported by Pei et al. [42] in largemouth bass, *Micropterus salmoides,* diseased with *A. veronii* in which there was necrotic and hemorrhagic lesions in the liver, there was infiltration by a large quantity of inflammatory cells into the kidney and mortification occurred in glomerulus, there was also hemosiderosis in the spleen of diseased fish. Tissue disintegration was possibly caused by the cytopathic effect of bacteriological toxins [42]. Hemosiderin buildup might be the outcome of blood cells destruction in the spleen where iron was unconfined from hemoglobin increasing hemosiderin [43]. Comparable histopathology was also detected in zebrafish, Nile tilapia, and cucian carp infected with *A. veronii* [21, 32, 33].

Therefore, all findings in this study provide important information on LD_50_, clinical signs, gross and microscopic lesions of *A. veronii* biovar *sobria* A4 strain which can help in coming up with treatment approaches for the control and prevention of this bacteriosis.

## 5. Conclusions and Recommendations

The LD_50_ of *Aeromonas veronii* biovar *sobria* A4 strain in Nile tilapia was determined to be 1.5 × 10^8^ CFU^−1^mL. Evident clinical signs induced by *A. veronii* biovar *sobria* A4 strain were observed in this study. These included skin darkening, erratic movements with loss of balance, anorexia, swimming at the bottom of the aquarium, lethargy and irregular breathing. The gross lesions included hemorrhages and ulceration on the skin, enlargement and hemorrhages on the liver, congestion and enlargement of the spleen, ascites and distention of gall bladder. Considerable number of mortalities especially in the high concentration of the bacterium was also observed. This shows that *A. veronii* biovar *sobria* A4 strain is pathogenic and is capable of causing massive losses in aquaculture. Even though the diseased fish with lower concentration of *Aeromonas veronii* biovar *sobria* A4 strain showed no clinical signs and death, in aquaculture, the radical environmental fluctuations, inappropriate management and parasitic contagions are pertinent aspects that might cause occurrences of bacteriological diseases. Hence, further research should be done on the pathogenicity of this bacterium to help come up with better mitigation measures to avoid its outbreak.

## Data Availability

Data used to support the findings of this study is available upon request.

## Supporting Information

Supporting Table 1: Mortality of challenged Nile tilapia during the LD50-96h of *A. veronii* biovar *sobria* A4 strain; Supporting Table 2: Frequency (%) of pathological lesions in assay of inoculation of Nile tilapia with *A*. *veronii* biovar *sobria* A4 strain; Supporting Table 3: Frequency of clinical sign and macroscopic lesion per dilution in the assay of inoculation of Nile tilapia with *A*. *veronii* biovar *sobria* A4 strain.
