# Supplementary material for: Lethal Dose, Clinical Signs, Gross and Microscopic Lesions Induced by Aeromonas veronii Biovar sobria A4 Strain in Experimentally Challenged Nile Tilapia (Oreochromis niloticus)
Source: Vet Med Int. 2025 Jun 12;2025:5525701. doi: 10.1155/vmi/5525701 (PMC12178767; doi:10.1155/vmi/5525701)
Supplement: Supporting Information — Additional supporting information can be found online in the Supporting Information section. [file 5525701.f1.docx]

**Supplementary File: Lethal dose, clinical signs, gross and microscopic lesions induced by *Aeromonas veronii* biovar *sobria* A4 strain in experimentally challenged Nile tilapia (*Oreochromis niloticus*)**

**Table S1: Mortality of challenged Nile tilapia during the LD_50-96h_ of *A. veronii* biovar *sobria* A4 strain**

| Bacterial concentration/Day | 1 | 2 | 3 | 4 | 5 | 6 | 7 | 8 | 9 | 10 | Survived | Died | Cumulative survived | Mortality rate (%) | Cumulative dead | Total |  | Cumulative mortality (%) |
| --- | --- | --- | --- | --- | --- | --- | --- | --- | --- | --- | --- | --- | --- | --- | --- | --- | --- | --- |
| 1.5x10^9^ | 6 | 1 | 0 | 0 | 0 | 0 | 0 | 0 | 0 | 0 | 3 | 7 | 3 | 70 | 18 | 21 |  | 85.71 |
| 1.5x10^8^ | 0 | 0 | 0 | 0 | 0 | 0 | 2 | 1 | 1 | 0 | 6 | 4 | 9 | 40 | 11 | 20 |  | 55 |
| 1.5x10^7^ | 0 | 0 | 0 | 0 | 1 | 0 | 0 | 1 | 1 | 0 | 7 | 3 | 16 | 30 | 7 | 23 |  | 30.43 |
| 1.5x10^6^ | 0 | 0 | 0 | 1 | 0 | 0 | 0 | 1 | 0 | 0 | 8 | 2 | 24 | 20 | 4 | 28 |  | 14.28 |
| 1.5x10^5^ | 0 | 0 | 1 | 1 | 0 | 0 | 0 | 0 | 0 | 0 | 8 | 2 | 32 | 20 | 2 | 34 |  | 5.88 |
| 1.5x10^4^ | 0 | 0 | 0 | 0 | 0 | 0 | 0 | 0 | 0 | 0 | 10 | 0 | 42 | 0 | 0 | 42 |  | 0 |
| 0 | 0 | 0 | 0 | 0 | 0 | 0 | 0 | 0 | 0 | 0 | 10 | 0 | 0 | 0 | 0 | 0 |  | 0 |

**Table S2: Frequency (%) of pathological lesions in assay of inoculation of Nile tilapia with *A veronii* biovar *sobria* A4 strain**

| Macroscopic lesions observed among the challenged fish | Frequency (%) |
| --- | --- |
| Erosion of the fins | 6.3 |
| Scale loss exposing the underlying skin at the base of caudal fin | 13.73 |
| Increased mucus on the skin surface | 13.8 |
| Inflamed vent | 13.8 |
| Congested and hemorrhagic gills | 15 |
| Darkening of the skin | 16.3 |
| Ascites | 16.3 |
| Exophthalmia | 17.5 |
| Distended gall bladder | 18.8 |
| Congestion of the operculum | 20 |
| Hemorrhages on the skin surface | 20 |
| Hemorrhages and enlargement of the liver | 21.3 |
| Enlargement and congestion of spleen | 22.5 |
| Cloudiness of the eye | 32.5 |

**Table S3**: **Frequency of clinical sign and macroscopic lesion per dilution in the assay of inoculation of Nile tilapia with *A. veronii* biovar *sobria* A4 strain**

| Clinical sign/macroscopic lesion | Frequency (%) per dilution (CFU/ml) | | | | | | |
| --- | --- | --- | --- | --- | --- | --- | --- |
|  | 0 | 1.5x10^4^ | 1.5x10^5^ | 1.5x10^6^ | 1.5x10^7^ | 1.5x10^8^ | 1.5x10^9^ |
| Darkening of the skin | 0 | 0 | 20 | 20 | 20 | 30 | 40 |
| Hemorrhages on the skin surface | 0 | 0 | 20 | 20 | 30 | 40 | 60 |
| Scale loss exposing the underlying skin at the base of caudal fin | 0 | 0 | 10 | 10 | 20 | 30 | 40 |
| Hemorrhages and enlargement of the liver | 0 | 0 | 20 | 20 | 30 | 40 | 70 |
| Enlargement and congestion of spleen | 0 | 0 | 20 | 20 | 30 | 40 | 70 |
| Distended gall bladder | 0 | 0 | 10 | 10 | 30 | 40 | 70 |
| Erosion of the fins | 0 | 0 | 0 | 0 | 10 | 20 | 30 |
| Ascites | 0 | 0 | 10 | 10 | 20 | 30 | 70 |
| Cloudiness of the eye | 0 | 0 | 20 | 30 | 50 | 70 | 90 |
| Exophthalmia | 0 | 0 | 10 | 10 | 20 | 40 | 70 |
| Increased mucus on the skin surface | 0 | 0 | 0 | 10 | 20 | 30 | 50 |
| Congestion of the operculum | 0 | 0 | 1 | 1 | 20 | 40 | 80 |
| Congested and hemorrhagic gills | 0 | 0 | 10 | 10 | 20 | 30 | 50 |
| Inflamed vent | 0 | 0 | 10 | 10 | 20 | 20 | 50 |
| Erratic movement with loss of balance | 0 | 0 | 40 | 60 | 80 | 80 | 80 |
| Anorexia | 0 | 20 | 50 | 70 | 70 | 90 | 90 |
| Lethargy | 0 | 10 | 40 | 50 | 60 | 80 | 90 |
| Swimming at the bottom of aquarium | 0 | 20 | 50 | 70 | 70 | 90 | 90 |
| Irregular breathing | 0 | 0 | 20 | 30 | 30 | 40 | 40 |
